# Serosurveillance of Coxiellosis (Q-fever) and Brucellosis in goats in selected provinces of Lao People’s Democratic Republic

**DOI:** 10.1371/journal.pntd.0006411

**Published:** 2018-04-12

**Authors:** Rebekah J. L. Burns, Bounlom Douangngeun, Watthana Theppangna, Syseng Khounsy, Mavuto Mukaka, Paul W. Selleck, Eric Hansson, Matthew D. Wegner, Peter A. Windsor, Stuart D. Blacksell

**Affiliations:** 1 Faculty of Veterinary Science, University of Sydney, Camden, Australia; 2 National Animal Health Laboratory, Department of Livestock and Fisheries, Ministry of Agriculture, Vientiane, Lao People’s Democratic Republic; 3 Mahidol-Oxford Tropical Medicine Research Unit, Faculty of Tropical Medicine, Mahidol University, Bangkok, Thailand; 4 Centre for Tropical Medicine & Global Health, Nuffield Department of Medicine, University of Oxford, Oxford, United Kingdom; 5 Laboratory Consultant, Geelong, Australia; 6 Laboratory Consultant, Creswick, Australia; 7 Armed Forces Institute of Medical Sciences, Bangkok, Thailand; 8 Lao-Oxford-Mahosot Hospital-Wellcome Trust Research Unit (LOMWRU), Mahosot Hospital, Vientiane, Lao People’s Democratic Republic; Walter and Eliza Hall Institute, AUSTRALIA

## Abstract

Goat raising is a growing industry in Lao People’s Democratic Republic, with minimal disease investigation to date, especially zoonoses. This study determined the proportional seropositivity of two zoonotic diseases: Q fever (causative agent *Coxiella burnetii*) and Brucellosis (*Brucella* species) in goats across five provinces (Vientiane Capital, Xayaboury, Xiengkhuang, Savannakhet and Attapeu). A total of 1458 goat serum samples were tested using commercial indirect ELISA for both pathogens, plus Rose Bengal agglutination test for Brucellosis. Overall individual seropositivity of *C*. *burnetii* was 4.1% and *Brucella* spp. was 1.4%. A multiple logistic regression model identified that province (Vientiane Capital, p = 0.05), breed (introduced Boer mixed breed, p = 0.006) and age (goats ≥3 years old, p = 0.014) were significant risk factors for *C*. *burnetii* seropositivity. The results of the survey indicated that province (Vientiane Capital, p<0.001), breed (introduced Boer mixed breed, p<0.001), production system (commercial, p<0.001), age (adult, p = 0.004), and farm size (large, 0.001) were all significant risk factors seropositivity for *Brucella* spp. It was concluded that Lao goats have been exposed to both *C*. *burnetii* and *Brucella* spp. however the risk of clinical disease has not yet been determined and there is an urgent need to determine human health risks and economic losses caused by Q fever and Brucellosis.

## Author summary

Goat raising is a growing industry in Lao People’s Democratic Republic however there is very little information whether or not goat raising poses a disease threat to farmers and the general population through diseases that may be transmitted between animals and humans (i.e., zoonotic diseases). To determine this, we tested goats for antibodies against two zoonotic diseases: Q fever (causative agent *Coxiella burnetii*) and Brucellosis (*Brucella* species) in Lao goats across five provinces (Vientiane Capital, Xayaboury, Xiengkhuang, Savannakhet and Attapeu). The presence of antibodies does not necessarily indicate active disease but that animals have been previously exposed to Q fever and Brucellosis. A total of 1458 goat serum samples were tested and the overall antibody positivity of the goats for *C*. *burnetii* was 4.1% and *Brucella* spp. was 1.4%. The highest risk of having Q fever antibodies was the goats being based in Vientiane Capital, of Boer mixed breed and ≥3 years old. The highest risk of having *Brucella* spp. antibodies was being based in Vientiane Capital, of Boer mixed breed as well as factors related to production system, age, and farm size. There is an urgent need to determine human health risks and economic losses caused by Q fever and Brucellosis.

## Introduction

Lao People’s Democratic Republic (Laos) is a landlocked country in the Greater Mekong Sub-region with an economy greatly dependent on agriculture [1]. Livestock have become increasingly important for improving rural livelihoods in Laos, providing a source of high quality protein, manure as fertiliser for plant growth, a means of household wealth storage, and income to buy food, education and healthcare [2].

Goats are becoming increasingly important for smallholder food farming in Laos [3, 4], providing livestock products that are perceived to require lower inputs than cattle and buffaloes. Furthermore, following regional economic growth there has been an increase in regional demand for goat meat in Vietnam and China, leading to rapidly increasing smallholder goat population and appearance of several commercial farms throughout Laos. Anecdotal reports suggest that there is no commercial milk or cheese production. The 2011 census reported that 45,000 farm households raised goats [3], however, it is difficult to report accurately on diversity of goat farming in Laos as it is the smallest livestock sector and is not always included in demographic reports. There is a need to focus on goat and goat farmer health in Laos as this has largely gone without investigation.

Human health is closely linked to livestock health. Healthy livestock can provide food, wealth and financial security, whereas unhealthy or diseased livestock cannot, and may be a reservoir for diseases infectious to humans (i.e., zoonoses). The close working relationship of farmers and their families with their animals allows for zoonotic disease transmission [1] with *Coxiella burnetii* (causing Q fever in humans) and *Brucella melitensis* considered potentially important bacterial zoonotic pathogens associated with goats in Laos [5]. Both agents can cause undulant fever and chronic disease in humans [5, 6]. These pathogens have the ability to cause large-scale outbreaks due to their low infectious dose, resistance in the environment and ability to travel via aerosolisation of the pathogens [5–7]. Q fever and Brucellosis are difficult to diagnose and treat in humans due to their non-specific presentation and intracellular nature [5, 7, 8]. Furthermore, *C*. *burnetii* and *Brucella* spp. can economically impact rural livelihoods as they reduce productivity due to reproductive loss in livestock herds [5, 8, 9]. *C*. *burnetii* and *Brucella* spp. are considered biothreats and classified as “Select Agents” in the USA [10, 11].

*C*. *burnetii* seroprevalence studies in Laos have revealed the pathogen is not widely distributed in cattle, despite the likelihood of an epidemiological hotspot in the Thailand-bordered province of Xayaboury [12, 13]. Thailand is a significant trading partner with Laos, and *C*. *burnetii* antibodies are reportedly present in 4% of Thai goats [14]. Surveys for *Brucella* spp. antibodies in Laos has revealed only a limited distribution of this pathogen in the Lao cattle herd [12, 13]. However, studies have revealed 1.4% of goats and 12.1% of goat herds in Thailand are seropositive for *Brucella* spp. [15]. Human Q fever and brucellosis case reporting is increasing in Thailand, with close contact with goat parturition material and consumption of raw goat meat considered as risk factors for contracting both diseases [14, 16]. One Thai study estimated that 12.6% of occupationally-exposed persons are seropositive for *C*. *burnetii* antibodies in selected provinces [14]. A report from 2004 outlines two Brucellosis cases, the first report in scientific literature since the 1970s, with one case having a history of drinking raw goat’s milk [17]. Following an outbreak of undulant fever in 2006 in a province northeast of Bangkok, village members were tested for exposure to Brucellosis of which 43.5% had antibodies to *B*. *melitensis*, with risk factors including recent contact with goats during their parturition and eating raw goat meat [16]. There are no overall human population seroprevalence or incidence estimates for Q fever or Brucellosis in Thailand available in the literature. This increase in reporting could be caused by increased disease awareness rather than an increase in disease incidence. Even though there are no reports of human Q fever or Brucellosis in Laos to date, this may be due to under-reporting rather than lack of disease manifestation, as there have been no specific investigations into these pathogens reported in the literature to date.

Considering the high risk of zoonoses due to factors including to the close working nature of farmers and livestock in Laos, porous borders, rapidly increasing smallholder goat population and appearance of commercial enterprises, it is important to investigate the prevalence of *C*. *burnetii* and *Brucella* spp. in Lao goats. This study reports on a serological survey of that aimed to determine the seroprevalence of *C*. *burnetii* and *Brucella* spp. antibodies in Lao goat herds in selected provinces and to identify potential risk factors associated with presence of infection. Furthermore, an understanding of infectious diseases in Lao goats enables development of disease prevention and control strategies and public health policies, supporting smallholder livestock farmers to increase their productivity, and therefore income, whilst minimizing human disease.

## Materials and methods

### Ethics statement

This study was conducted in compliance with State Acts and National Codes of Practice for Ethical Standards, with animal and human ethics approval obtained from The University of Sydney Ethics Committee (project no. 2015/765 and 2014/783, respectively). Verbal consent was obtained from all of the goat owners prior to the collection of the sample.

### Study sites

**Fig 1:**
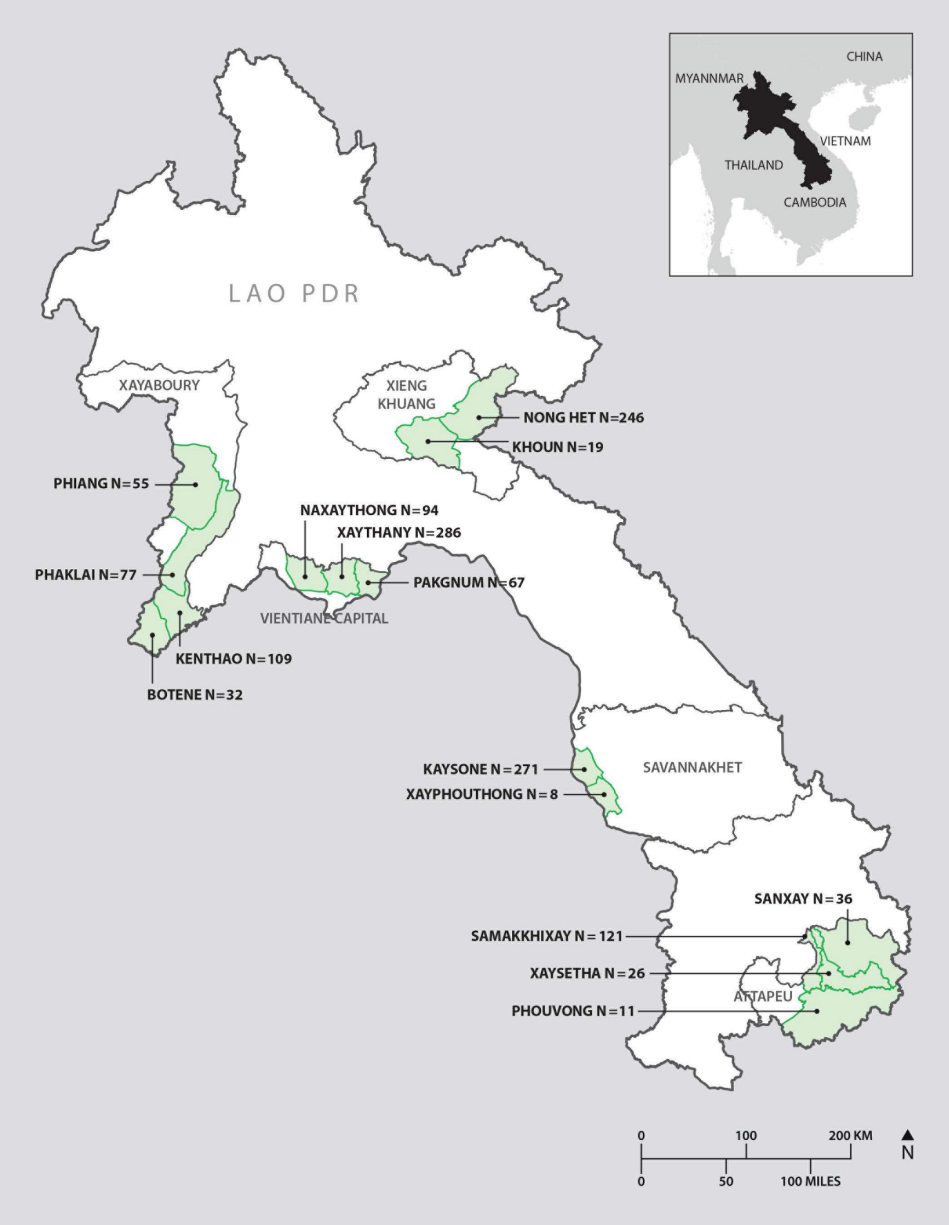
Map of Laos and districts sampled in this study. Numbers of samples collected in each location is indicated.

This study was conducted in five provinces in Laos: Vientiane Capital, Xayaboury, Xiengkhuang, Savannakhet and Attapeu (Fig 1). Samples were collected between October 2016 and May 2017. Vientiane Capital was selected due to the emergence of commercial goat enterprises and the convenience of proximity to the National Animal Health Laboratory (NAHL). Xayaboury was selected as a follow up to a previous study suggesting there was an epidemiologic “hot spot” for Q fever in cattle located in districts bordering Thailand [12, 13]. Samples were collected from Xiengkhuang, Savannakhet and Attapeu as part of necropsy training workshops run by the same team.

To provide some guidance regarding the number of samples to be collected, sample size was calculated using the formula: *n = (Z*^*2*^ *P(1-P))/e*^*2*^, where *Z* is the value from a standard normal distribution corresponding to the desired confidence level, *P* is the expected true proportion and *e* is the desired precision. A sample size to estimate individual level seroprevalence with a precision of 0±1% with an expected herd prevalence of 3.5% and confidence level of 95% was calculated using “AusVet Epitools” website [18]. As no goat seroprevalence studies for *C*. *burnetii* and *Brucella* spp. had been performed in Laos previously, estimated herd prevalence was extrapolated from caprine *C*. *burnetii* seroprevalence studies in neighbouring Thailand [14]. In 2011, the Lao agriculture census estimated that there were 215600 goats in Laos [3]. This resulted in a target sample size of 1291 goats however this was an estimate for the whole of Laos rather than selected provinces/districts. Using this sample size guidance, a total of 69 villages within 15 districts were selected (Fig 1). Districts were chosen with close assistance from staff from the Department of Livestock and Fisheries in Laos (DLF). Districts were visited if they had the following criteria: accessible via road vehicle; close working relationship with DLF staff; and households or farms that raised goats. Upon arrival at each district, the sampling team consulted provincial and district staff as to which villages raised goats. The sampling team visited participating different households and farms within each village following discussions between the sampling team, DLF staff, village chiefs and farmers. The team aimed to sample as many goats as possible within each village and household, this included every goat presented to the team.

### Sample collection

Blood (3–5ml) was collected from the jugular vein into a sterile syringe and allowed to clot. The serum was removed and stored at 4°C for transport back to NAHL where it was centrifuged at 5000 rpm for 5 minutes and stored at -80°C until further analysis. Samples collected in 2017 obtained epidemiological data including date of collection, owner name, age via examination of teeth [19] and gender. Additionally, for the provinces of Vientiane Capital, Xayaboury and parts of Attapeu, breed (native Kambing-Katjang or introduced Boer/ Boer mixed), the number of goats per household owned, and production system (commercial enterprise with employed persons to raise goats or small holder, family raised household goats) were recorded. However, no such data was recorded for the 704 samples collected in 2016.

### Serological testing

#### Q fever ELISA

Sera were tested for whole antibodies against *C*. *burnetii* inactivated phase I and II antigen using a semi-quantitative, indirect commercial ELISA test (CHECKIT Q fever Antibody ELISA Test Kit, ID-VET, product code FQS-MS-5P, ID-Vet, Gabrels, France) according to the manufacturer’s instructions [20]. Results were expressed as a percentage of the optical density (%OD) reading of the test sample calculated as %OD = 100 * (S-N)/(P-N), where S, N and P are the values of the sample (S) and OD of negative (N) and positive (P) controls, respectively. Samples with %OD ≥40% were considered positive.

#### *Brucella* spp. ELISA and Rose-Bengal

Sera were tested for whole antibodies against *Brucella* spp. using an indirect multi species commercial ELISA test kit (ID-Screen Brucellosis Serum Indirect Multispecies, ID-VET, product code BRUS-MS-10P, Gabrels, France) according to the manufacturer’s instructions [21]. Results were expressed as a percentage of the optical density (%OD) as described above. Samples with %OD≥120% were considered positive. Positive sera were then tested for *Brucella* antigen further using a Rose-Bengal *Brucella* agglutination test, with 30μl of serum was combined with 30μl of Rose-Bengal reagent and mixed on a glass plate for 4 minutes and the development of agglutination was assessed. Samples that agglutinated before 4 minutes were considered positive. A sample was considered serologically positive for *Brucella* spp. antibodies if the sample was above the cut off for the ELISA and had a positive Rose-Bengal result.

### Statistical analysis

Data was entered into Microsoft Excel and analysed using Stata/SE version 15.0 for Macintosh (StataCorp, College Station, TX). For both pathogens, serological prevalence was calculated as the proportion of animals that had detectable antibodies in the same population, with 95% confidence intervals. For both *C*. *burnetii* and *Brucella* spp., measures of association for categorical data were assessed using either Pearson’s Chi-squared test or Fisher’s exact test as appropriate.

For calculations regarding *C*. *burnetii* seropositivity only, univariable logistic regression models were fitted to obtain unadjusted estimates of odds ratios (OR). Furthermore, a multivariable logistical regression model was performed to determine factors independently associated with *C*. *burnetii* seropositivity. Only data with full epidemiological information (754 samples) were included in this model, as the remaining samples were collected prior to 2017 without full epidemiological and clinical information. Variables with univariable significance (p≤0.05) were entered into the multivariable model. All tests of significance were performed at 5% level of significance.

## Results

### Distribution of animals

The distribution of provinces, districts and villages sampled are outlined (Fig 1, Table 1). The number of serum samples collected per village ranged from 2 to 80, with a median of 19.

**Table 1:** Distribution of goats tested throughout Lao PDR.

Province	Districts	Villages	n
Attapeu	4	6	194
Savannakhet	2	15	279
Vientiane Capital	3	12	447
Xayaboury	4	11	273
Xiengkhuang	2	23	265
Total	15	67	1458

### Seroprevalence of *C*. *burnetii* antibodies

A total of 1458 goats were sampled. Overall, 60/1458 (4.1%; 95% CI 3.0, 5.0) of goats sampled were seropositive for *C*. *burnetii* antibodies (Table 2). Notably, the OR of individual goat *C*. *burnetii* seropositivity within Vientiane Capital was 33.4% (95% CI 4.6, 243.7, p = 0.001) and in Xayaboury was 8.4% (95% CI 1.0, 67.6, p = 0.046) respectively, with both statistically significant compared to the chosen reference province of Savannakhet (Table 2).

**Table 2:** Results of univariable and multivariable logistical regression on *C*. *burnetii* seropositivity.

			Univariable analysis		Multivariable analysis	
Parameter	n	% (95% CI^a^)	OR (95% CI)	p	OR (95%CI)	p
Total population sampled	1458	4.1 (3.0, 5.0)				
Province						
Savannakhet	279	0.4 (0.0, 2.0)	1.0 (reference)		N/A	
Attapeu	194	1.6 (0.5, 3.7)	4.4 (0.5, 42.3)	0.203	0.8 (0.5, 11.17)	0.244
Xayaboury	273	2.9 (1.3, 6.0)	8.4 (1.0, 67.6)	0.046	1 (reference)	
Vientiane Capital	447	10.7 (8.0, 14.0)	33.4 (4.6, 243.7)	0.001	5.4 (1.0, 29.32)	0.05
Xieangkhuang	265	0.0 (0.0, 1.3^b^)	N/A	N/A	N/A	
Breed						
Native	480	1.6 (0.7, 3.3)	1.0 (reference)		1.0 (ref)	
Introduced Boer	340	13.5 (10.1, 17.6)	9.2 (4.3, 19.8)	<0.001	6.9 (1.74, 27.27)	0.006
Production System						
Small holder	340	2.5 (1.3, 4.3)	1.0 (reference)		1.0 (reference)	
Commercial	480	12.4 (9.0, 16.3)	5.5 (2.8, 10.6)	<0.001	1.0 (0.2, 5.0)	0.981
Age category (years)						
Kids (<1)	336	2.1 (0.8, 4.2)	1 (reference)		1 (reference)	
Young adults (1–2)	439	2.3 (1.1, 4.1)	1.1 (0.4, 2.9)	0.855	1.1 (0.3, 12.5)	0.895
Older adults (≥3)	399	10.5 (7.7, 13.9)	5.5 (2.4, 12.5)	<0.001	4.06 (1.3, 12.5)	0.014
Gender						
Male	267	1.5 (0.04, 2.96)	1 (reference)		1 (reference)	
Female	1176	4.7 (3.5, 6.0)	3.2 (1.2, 9.0)	0.025	2.5 (0.5, 11.2)	0.244
Farm Size						
Small (<15)	137	3.7 (1.2, 8.3)	1 (reference)		1 (reference)	
Medium (15–40)	253	2.8 (1.1, 5.6)	0.8 (0.2, 2.4)	0.6	0.3 (0.0, 0.5)	0.143
Large (>40)	359	11.7 (8.5, 15.5)	3.5 (1.4, 9.0)	<0.001	0.59 (0.1, 2.3)	0.461

a Confidence interval

b one sided, 97.5% Confidence Interval

Within Vientiane Capital, all districts had some seropositive animals (Table 3) although there was a significant difference in seroprevalence between the districts sampled in Vientiane Capital (p<0.001), with the highest seroprevalence in Parkgnum (25.4%) (Table 3). Vientiane Capital had 75% (8/12) villages with a seropositive result, with significant difference between villages (p<0.001) including Ponsavan village with the highest seropositivity of 29.6% (Table 3). Within Xayaboury, Kentao (7.3%) was the only district to display any seropositivity.

**Table 3:** *C*. *burnetii* and *Brucella* spp. seropositivity by district and village within Vientiane Capital.

District	Villages	n	*Coxiella burnetii* antibody ELISA			*Brucella* antibody ELISA + RB^a^		
			%	95% CI^b^	p	%	95% CI^b^	p
Naxaythong		94	8.5	3.7, 16.1		8.5	3.7, 16.1	
	Hongngoua	24	22.6	9.5, 41.1		22.6	9.6, 41.1	
	Nagnang	32	3.0	0.0, 15.8^c^		3.0	0.0, 15.7	
	Sisavat	20	0.0	0.0, 16.8^c^		0.0	0.0, 16.8^c^	
	E lai	10	0.0	0.0, 30.8^c^	0.014^e^	0.0		0.014^e^
Xaythany		286	8.0	5.1, 11.8		3.4	1.7, 6.3	
	Douagboutdi	70	8.6	3.2, 17.7	<0.001^e^	0.0	0.0, 5.1^c^	<0.001^e^
	Hai	8	12.5	0.3, 52.7		0.0	0.0, 36.9^c^	
	Lartkhoay	26	0.0	0.0, 13.2^c^		0.0	0.0, 13.2^c^	
	Nathae	80	5.0	1.4, 12.3		0.0	0.0, 4.5^c^	
	Phailom	49	0.0	0.0, 7.3^c^		0.0	0.0, 7.3^c^	
	Ponsavan	27	29.6	13.8, 50.2		37.04	19.4, 57.6^c^	
	Thongmang	26	15.4	4.4, 34.9		0.0	0.0, 13.2^c^	
Parkgnum		67	25.4	15.5, 37.5		0.0	0.0, 5.4^c^	
	Naxon	67	25.4	15.5, 37.5		0.0	0.0, 5.4^c^	
p value districts in Vientiane Capital					<0.001^d^			0.002^e^
p value villages in Vientiane Capital					<0.001^e^			<0.001^d^

a Rose Bengal agglutination test

b Confidence Interval

c one sided 97.5% confidence interval

d Pearson’s Chi Square test

e Fisher’s exact test

Where full epidemiological information was available (n = 744; 51%), univariable and multivariable logistic regression was performed. The OR for Boer crossbred goat seropositivity for *C*. *burnetii* was significantly greater when compared to native Kambing-Katjang goats (OR 9.2; 95% CI 4.3, 19.8, p<0.001) (Table 2). The OR for goats sampled from commercial farms for *C*. *burnetii* seropositivity was significantly greater than goats sampled from smallholder farms (OR 5.5; 95% CI 2.8, 10.6, p<0.001) (Table 2). The OR for goats sampled from large farms indicated *C*. *burnetii* seropositivity was significantly greater when compared to other farms (OR 3.5; 95% CI 1.4, 9.0, p<0.001) (Table 2). The likelihood of seropositivity increased with age, with antibodies detected in 10.5% of goats ≥3 years of age when compared with only 2.3% of young adults (ages 1–2) and 2.1% of kids (aged <1). The adult goats (aged >3 years) had significantly increased exposure to *C*. *burnetii* than kid goats (OR 5.5; 95% CI 2.4, 12.5, p<0.001) (Table 2). However, there was no statistical difference between the young adult goats (aged 1–2) and the kid goats, (OR = 1.1; 95% CI 0.4, 2.9, p = 0.855). Overall, age was a significant variable in the univariable analyses, p = 0.014 (Likelihood ratio test, not in the table).

All variables (province, breed, production system, age category, gender and farm size) were significantly associated with *C*. *burnetii* seropositivity on univariable analysis (Table 2). There was a significant difference between genders (p = 0.025) with 4.7% of female goats being seropositive and only 1.5% of male goats demonstrating seropositivity (Table 2). Subsequently, all variables were included in the multivariable analysis. The following variables had multivariable significance: introduced Boer breed goats (OR = 6.9; 95% CI 1.7, 27.3, p<0.001); goats 3 years or older (OR 4.1; 95% CI 1.3, 12.5, p = 0.004); and goats located in Vientiane Capital had marginal significance (OR 5.4; 95% CI 1.0, 29.3, p = 0.05) (Table 2).

### Seroprevalence of *Brucella* spp.

Overall, 20/1458 (1.4%; 95% CI 0.8, 2.2) goats tested demonstrated *Brucella* spp. seropositivity having serial positivity to both ELISA and Rose-Bengal agglutination tests (Table 4), despite 3.0% of goat samples returned seropositive ELISA results alone. Significant differences were noted between provinces (Pearson’s chi^2^ p<0.001), with the highest seroprevalence noted in Vientiane Capital (4.0%), and in Attapeu (1.6%). *Brucella* spp. seropositivity was not detected from Xayaboury, Savannakhet or Xiengkhuang (Table 4).

**Table 4:** *Brucella* spp. seropositivity distribution and contingency table.

	*Brucella* antibody ELISA + Rose-Bengal			
Parameter	n	%	95% CI^a^	p
Total population sampled	1458	1.4	0.8, 2.2	
Province				
Attapeu	194	1.6	0.3, 4.4	<0.001^c^
Savannakhet	279	0.0	0.0, 1.3^b^	
Xayaboury	273	0.0	0.0, 1.3^b^	
Vientiane Capital	447	4.0	2.4, 6.2	
Xiengkhuang	265	0.0	0.0, 1.3^b^	
Breed				
Native	480	0.0	0.0, 0.8^b^	<0.001^c^
Introduced Boer	340	5.6	3.3, 8.5	
Production System				
Small holder	340	0.0	0.0, 0.8^b^	<0.001^c^
Commercial	480	5.6	3.4, 8.6	
Age category (years)				
Kids (<1)	336	0.3	0.0, 1.6	0.004^c^
Young adults (1–2)	439	1.1	0.4, 2.6	
Older adults (≥3)	399	3.3	1.7, 5.5	
Gender				
Female	1176	1.5	0.9, 2.4	0.23^c^
Male	267	0.4	0.0, 2.0	
Farm size				
Small < 15	137	0.0	0.0, 2.6^b^	
Medium (15–40)	253	0.0	0.0, 1.4^b^	
Large (>40)	359	5.3	3.2, 8.1	<0.001^c^

a Confidence interval

b One sided, 97.5% Confidence interval

c Fisher’s Exact Test

There was a significant difference of seropositivity to *Brucella* spp. between districts sampled in Vientiane Capital (p = 0.009), with the highest seroprevalence in Naxaythong (9.5%), followed by Xaythany (3.4%) (Table 3). Parkgnum district had no *Brucella* spp. positive samples. Within Vientiane Capital, only three (25.0%) villages were seropositive for *Brucella* spp: Ponsavan village (37.0%), Hongngua (22.6%), and Nagnang (3%) (Table 4). This finding was significant compared with other villages within Vientiane Capital (Fisher’s exact p<0.001). Within Attapeu province, there was a significant difference in seropositivity detected between districts (Fisher’s exact p = 0.014), with 2/9 (18.1%) animals seropositive in Phouvong and 1/120 (0.8%) of animals seropositive in Sahmakisai. The other two districts within Attapeu had no *Brucella* seropositive animals.

Where full epidemiological information was (n = 754) available, there was a significant difference demonstrated between goat breeds (p<0.001), with 5.6% of introduced Boer or Boer cross breeds found positive, and no seropositivity detected in native breeds (Table 4). There was a significant difference demonstrated between production systems (p<0.001), with 5.6% of goats on commercial farms seropositive for *Brucella* spp. and no seropositivity detected in smallholder systems (Table 3). Animals were more likely to have positive antibodies detected if they were sampled on a large farm with over 40 animals (5.3%), with no seropositivity detected on farms with 40 or fewer goats (p<0.001).

There was a significant difference in age of the goats (p<0.001), where adults ≥3 years recorded the highest proportion of seropositivity (3.3%) followed by goats 1–2 years old (1.1%) (Table 4). Only 0.3% of kids aged <1 year old was found to be seropositive. There was no association detected between genders (p = 0.23) (Table 3).

## Discussion

This study investigated the presence of exposure of Lao goats to zoonotic pathogens, *C*. *burnetii* and *Brucella* spp, and determined their seropositivity. The seroprevalence of both *C*. *burnetii* and *Brucella* spp. was much higher and more widespread in goats compared with previous cattle seroprevalence studies within Laos [12, 13]. Similar results were found in Thai goats for *C*. *burnetii* seroprevalence (4%)[14] and *Brucella* spp. seropositivity (1.4%), although *Brucella* spp. seropositivity appears more widespread in Thailand (12.1% of herds) [15]. The *Coxiella* study in Thailand utilised a similarly prepared ELISA from a different company (IDEXX) to this study however samples were also taken using convenience methodology so comparability of seroprevalence results between studies is limited [14]. The *Brucella* study utilised compliment fixation, Rose-Bengal and ELISA and if any test were positive the animal was considered positive [15] which may have artificially increased seropositivity when compared to the study presented here.

The results presented here clearly demonstrate a spatial difference of *C*. *burnetii* seropositivity with Vientiane Capital having significantly higher individual goat seropositivity than other provinces. Similar to previous studies, a hotspot of *C*. *burnetii* seroprevalence was demonstrated in cattle in Xayaboury province, located on Laos-Thailand border [12, 13]. Furthermore, goats in Vientiane were much more likely to be exposed to *Brucella* spp. than goats in other areas. This spatial distribution has not previously been reported for either pathogen in Laos, although is probably associated with Vientiane Capital being a major thoroughfare for international trade and the location of emerging commercial goat enterprises. In other global seroprevalence surveys for both pathogens, areas with high international trade are considered high risk for exposure to *C*. *burnetii* and *Brucella* spp. [22, 23]. It is a known problem that illegal movement of animals occurs throughout South East Asia through “porous borders” as demonstrated through Foot and Mouth disease outbreak studies [24]. It is possible that goats brought into Laos from other counties were already exposed to the pathogens, or that travel and intensification of production has contributed to possible infection. Nevertheless, there appears a significant public health risk to goat farmers and possibly consumers within Vientiane Capital in Laos, as recently identified with Orf virus infection [4].

It is interesting that for both pathogens, introduced Boer mixed bred goats were significantly more likely to be seropositive than native Kambing-Katjang goats, with *Brucella* spp. seroprevalence only reported in the introduced goats. Reasons for *C*. *burnetii* being higher in Boer mixed bred goats may be that these animals tended to be more intensively raised and on commercial farms and were likely to have been or descendants of imported animals. Intriguingly, resistance in native goats to *Brucella* spp. has been previously suggested with seroprevalence studies in Mexico [25] and Malaysia [22] reporting increased *Brucella* spp. exposure in imported breed goats compared to native animals. It has been suggested that different breeds of cattle may also be resistant to *Brucella* spp. infection through genetic innate immunity [26, 27]. Further studies are necessary to determine the possible role of genetics of goat immunity to a variety of pathogens.

Age was independently associated with seropositivity of *C*. *burnetii*, and there was a significant difference between age groups as risk for *Brucella* spp. seropositivity. Adult animals ≥3 years were more likely to be exposed to both pathogens, a finding consistent with literature and likely due to increasing opportunities of pathogen exposure [5, 28]. Similarly, female goats were more at risk of having antibody titer against *C*. *burnetii* than male goats, likely representing to the tropism for both pathogens to the placenta and mammary lymph nodes [6, 8].

Results demonstrated here indicating risk factors for infection in Laos included commercialization in comparison with smallholder systems and association of larger farms with higher seroprevalence are in contrast with studies in Thailand and elsewhere [15, 29, 30]. It is thought that smallholder farmers with free ranging goats were at higher risk for *Brucella* spp. exposure as the mobility of wandering herds favours spread of infectious disease when allowed to mix with naïve herds [15, 29, 30]. Intensification has been reported as a risk factor in seroprevalence studies [25, 31], where close contact within herds may also favour pathogen spread. Although herd size and commercialisation were significant risk factors on univariable analysis for *C*. *burnetii* seroprevalence, neither variable was significant on multivariable analysis and hence were potential confounding variables, despite the likelihood that these herds were introduced to infection before or after importation.

To accurately estimate prevalence of a disease in the population, sensitivity and specificity of a test must be known to approximate the occurrence of false results. The ID-Vet Q fever iELISA has been determined at 100% sensitivity and 100% specificity for Coxiellosis in cattle located in France, however no sensitivity and specificity reports have been performed for small ruminants [25, 31] so these estimations may not be accurate for small ruminant studies. Furthermore, as the status of a disease (endemic or not endemic) in a population can alter the sensitivity and specificity of a test in a region, analysis of sensitivity and specificity is required in Laos, and South East Asia as a whole.

Internal company testing of the ID-VET Brucella iELISA found 100% specificity on a herd of 160 goats in France, and 100% sensitivity on 5 goats in Southern Italy [21]. The small sample size brings into question the reliability of the estimations, which additionally might not be applicable to the region of South East Asia as sensitivity and specificity, can differ by regions [32, 33]. The Rose-Bengal test has been assessed at 94% sensitive and 99% specific [34] yet there is no estimation of sensitivity or specificity for the combined tests specific to the region. Latent class analysis can be used to determine the sensitivity and specificity of tests in the absence of a gold standard test for the region. Furthermore, it can be estimated using latent class analysis which test is most suitable, in what order it should be performed, and also if the tests should be utilised in parallel (all positives are included), or serially (only positives on all tests included, as in this study). For example, studies of cattle serology in Zambia indicated a competitive-ELISA paired with Fluorescent Polarisation test results in the highest sensitivity and specificity [35], and for sheep in Europe the blocking-ELISA is most accurate [36], and finally Rose-Bengal plus competitive ELISA test gave the best results for cattle testing in Zimbabwe [37]. However, it is a weakness of this study that this analysis has not been performed to determine the utility of testing in parallel or serially.

*Brucella* spp. and Q fever serology results must be interpreted in the context that Lao goats are not vaccinated for either disease, nor are there active control programs and as such it is highly unlikely that any of the positive serology results were attributable to local vaccine strains. Nevertheless, it is a limitation of this study that the farmers were not queried about vaccination. A positive *Brucella* spp. serology result can be caused by a cross-reaction with a range of bacterial species including *Yersinia* spp. giving rise to results that may not be fully accurate [38] although repeating tests can increase the reliability of results [34]. It is possible that the use of serial testing with Rose-Bengal and ELISA for *Brucella* antibodies reduced the number of sites with *Brucella* seropositivity than would have been reported by ELISA alone. Furthermore, following discussions with the ELISA kit manufacturers, a decision was made to classify "suggestive" positive samples as negative thereby decreasing the possibility of false-positive results. While the decision to employ serial ELISA and Rose-Bengal in this study was based on methodologies of previous *Brucella* spp. studies within Laos [12, 13], there is potential for latent class analysis to be utilised on this data set to determine the best combination of tests for the most appropriate sensitivity and specificity result in Laos.

In a preliminary investigation during this study, vaginal swabs were collected from goats located within Vientiane Capital, with one found to be positive for *Brucella* spp. DNA with real time PCR and none to have *C*. *burnetii* DNA (personal communication, Dr. Reka Kanitpun). This preliminary finding indicated that farmers and their families were at potential risk for contracting Brucellosis from their goats, although further investigations are needed to understand shedding patterns and speciation of *Brucella* spp., preferably using molecular diagnostic tools. This study did not differentiate the species of *Brucella* spp. as both the serological tests utilised were genus specific only. Goats are generally associated with *B*. *melitensis*, the species that is most pathogenic to humans, yet infection with *B*. *abortus* or *B*. *suis* is also common [5].

Although this present seroprevalence data demonstrates previous exposure of animals to these pathogens, serology alone does not provide a complete picture of infection status within an animal population. Seropositivity does not indicate disease manifestation, current shedding of pathogens, or consequently current risk of transmission. Studies have suggested that a significant proportion of animals that shed *C*. *burnetii* or *Brucella* spp. are not seropositive; furthermore animals can be seropositive and not be shedding [5, 6]. Furthermore, the proportion of animals shedding *C*. *burnetii* is independent of abortion history in a herd, and shedders might represent clinically unapparent infections [9, 39].

Outbreaks of human Q fever and Brucellosis are commonly linked to seasonal parturition in small ruminant production system [5, 9, 40, 41]. In developing nations, many fevers presenting to medical clinics go undiagnosed due to their general “ill-thrift” nature [42, 43]. It is imperative that medical practitioners in Laos are aware of Q fever and Brucellosis as differential diagnoses, especially for at risk populations, including livestock farmers with recent exposure to animal parturitions, pregnant women and people consuming raw goat products.

Control of zoonotic and transboundary disease pathogens proves difficult in developing nations where veterinary support and resource may be limited or unavailable. Vaccination of animals for either *C*. *burnetii* or *Brucella* spp. is not recommended as it must be given according to parturition calendars. Furthermore, vaccination will only reduce but not stop shedding, can cause goat abortions if given at incorrect times and can cause human disease if self-inoculated [5, 40, 42]. Test and culling is recommended for *Brucella* spp. seropositive farms and may have a role in this study where very few villages were considered likely to be affected. It is acknowledged this is currently difficult politically and financially. Disinfecting farms quarterly may reduce disease spread for both pathogens[22] although may not be applicable to rural village settings with free ranging goats.

Despite these issues, this study has addressed important knowledge gaps on *C*. *burnetii* and *Brucella* spp. seroprevalence in Lao goats whilst raising a number of other questions. Further investigations of the potential risk factors for transmission of the different species and farming practices are necessary to determine why Boer crossbred goats have higher seroprevalence. Further studies investigating shedding of both *C*. *burnetii* and *Brucella* spp. are required for speciation and potential trace back of disease transmission, plus collection of caprine placentas for PCR and pathology. There is urgent need to determine current Q fever and Brucellosis seroprevalence and occurrence of the diseases in humans, especially in at -risk populations including livestock farmers, others exposed to goat effluent, plus people consuming raw goat meat or milk products [17]. With the increasingly important contribution of goats to Lao and regional food security, the zoonotic issues from Lao production systems will very likely become increasingly important. International aid groups and commercial farms are advised to serologically test goats prior to importing them into Laos, and work closely with Lao veterinary services to ensure limited pathogen spread occurs both between villages and from animals to humans.

## Supporting information

S1 Dataset Goat sample data. (XLSX)
